# Drilling Machinability of Glass, Basalt, and Hybrid Epoxy Composites: Thrust Force, Thermal Load, and Hole Quality

**DOI:** 10.3390/polym17121643

**Published:** 2025-06-13

**Authors:** Eser Yarar, Mehmet İskender Özsoy, Sinan Fidan, Satılmış Ürgün, Mustafa Özgür Bora

**Affiliations:** 1 Department of Mechanical Engineering, Faculty of Engineering, Kocaeli University, Kocaeli 41380, Türkiye; eser.yarar@kocaeli.edu.tr; 2 Department of Mechanical Engineering, Faculty of Engineering, Sakarya University, Sakarya 54050, Türkiye; 3 Department of Airframe and Powerplant Maintenance, Faculty of Aeronautics and Astronautics, Kocaeli University, Kocaeli 41001, Türkiye; sfidan@kocaeli.edu.tr (S.F.); ozgur.bora@kocaeli.edu.tr (M.Ö.B.); 4 Department of Aviation Electrics and Electronics, Faculty of Aeronautics and Astronautics, Kocaeli University, Kocaeli 41001, Türkiye; urgun@kocaeli.edu.tr

**Keywords:** hybrid glass–basalt laminates, drilling machinability, thrust force, thermal response, hole quality, high temperature

## Abstract

The drilling machinability of glass fiber G_14_, basalt fiber B_14_, and two hybrid laminates (B_4_G_6_B_4_, G_4_B_6_G_4_) was evaluated through 36 full-factorial experiments employing an HSS-G drill, three spindle speeds (715, 1520, 3030 rpm), and three feed rates (0.1–0.3 mm rev^−1^). Peak thrust force varied from 65.8 N for B14 at 0.1 mm rev^−1^ to 174.3 N for G_14_ at 0.3 mm rev^−1^; hybrids occupied the intermediate range of 101–163 N. Infra-red thermography recorded maximum drill temperatures of 110–120 °C for G_14_, almost double those of B_14_, while both hybrids attenuated hotspots to below 90 °C. ANOVA attributed 73.4% of thrust force variance to feed rate, with material type and spindle speed contributing 15.5% and 1.7%, respectively; for temperature, material type governed 41.5% of variability versus 17.0% for speed. Dimensional quality tests revealed that the symmetric hybrid G_4_B_6_G_4_ maintained entrance and exit diameters within ±2% of the nominal 6 mm, whereas B_4_G_6_B_4_ over-expansion exceeded 8% at the lowest feed and G_14_ exit diameters grew to 6.1 mm at 0.3 mm rev^−1^. Integrating basalt compliance with glass stiffness, therefore, halves thrust force relative to G_14_, suppresses delamination and overheating, and offers a practical strategy to prolong tool life and improve hole quality in multi-material composite structures. These insights guide parameter selection for lightweight hybrid composites in aerospace, renewable-energy installations, and marine components worldwide.

## 1. Introduction

The polymer matrix–fiber composite materials have been investigated extensively for their use in structural applications because of their excellent specific strength, stiffness, fatigue life, low density, and superior corrosion resistance [1,2,3,4,5,6]. Although advanced manufacturing techniques can produce near-net-shaped FRP components, secondary drilling is often necessary for assembly. While unconventional methods like water jet cutting, laser machining, and ultrasonic-assisted cutting have been explored, conventional mechanical drilling remains the predominant method for hole-making in FRPs due to its practicality and widespread industrial use [7].

The most widely applied machining process in composite laminates is drilling, mainly to assemble structures. Drilling is impacted by several parameters in commercial composites, with effects both on efficiency and the possibility to cause damage to the material. The tool type, cutting speed, feed rate, and drilling methods are essential factors to determine the hole quality and structural stability [8,9,10]. Composite parts are usually produced near the desired shape to reduce machining, but finishing processes like edge trimming and finishing of holes and slot regions are still unavoidable. Drilling causes the top and bottom surfaces of holes to be damaged, generally measured by the measure of delamination or damage factor. Research data on woven glass fabric composites show that exit delamination is constant at increased feed rates. Owing to the poor thermal conductivity of polymeric matrix materials, heat will be generated, promoting tool wear and roughness at the surface [11].

The potential of natural fiber-reinforced composites to replace traditional fiber-reinforced petroleum-based composites is being thoroughly investigated. Various biomaterials are used to extract various natural fiber compositions. These bio composites are extensively used in numerous vital applications. When combining natural fibers with synthetic fibers, structures gained typically offer superior properties than natural fiber-reinforced composites [12,13]. Natural fibers and their composites are ideal for various industrial purposes owing to their low density, lightweight nature, and various special properties. Compared to synthetic fibers, which are predominantly produced as a byproduct of the maneuverability exhausting crude oil-refining process, natural fiber extracts are extremely cost-effective [14,15].

Every developed product’s material needs to be changed on a regular basis to make it more durable and user-friendly. When employed as reinforcement in composites, single natural or synthetic fibers have drawbacks such as lower mechanical strength, less durability, and increased cost. Hybrid composites can be used to overcome these drawbacks. Patel et al. [16] used isophthalic polyester resin as a matrix material and basalt and e-glass fibers as reinforcement to create hybrid fiber-reinforced composites. Three reactions, namely thrust force (TF), delamination at the top surface (DFentry), and delamination at the bottom surface (DFexit), were analyzed after drilling investigations were conducted on the designated composite samples by varying input parameters (stacking sequence, tool geometry, speed, and feed). ANOVA was utilized to determine the machining parameter’s greatest influence and percentage contribution, while the design of experiment (DOE) was utilized for combination details. Gray relational analysis (GRA) was employed to get the optimal results. In order to reduce delamination by minimizing thrust force, Murthy et al. [17] aimed at optimizing carbon–basalt hybrid composites’ drilling processes. With a fiber weight fraction of 50% and thickness of 6 mm, the composites were fabricated by manual lay-up. Following the design of experiments by Taguchi, experiments were conducted by performing drilling with three process factors: spindle speed of 750 rpm, 1000 rpm, 1250 rpm; feed rate of 50 mm/min, 75 mm/min, 100 mm/min; and drill diameter of 6 mm, 8 mm, 10 mm. As per the ANOVA results, the feed rate has the maximum effect over thrust force, followed by drill diameter, and spindle speed has the minimum effect. Sathish and Varadhan [18] discussed the influence of drilling parameters and filler weight content over delamination and surface roughness in hybrid basalt/glass fiber-reinforced polymer (HBGFRP) nanocomposites filled with multi-walled carbon nanotubes (MWCNTs) and nano-silica (SiO_2_) at 0%, 1%, and 2% weight content. The manual lay-up and compression molding fabricated the nanocomposites with a 12-layer stacking sequence. Box–Benkhen’s design of the response surface methodology optimized the spindle speed, feed rate, drill diameter, and filler content. The results were 1.0 wt.% MWCNTs + SiO_2_ reduced the delamination by 24%, with the minimum delamination factor of 1.29. The spindle speed ramp-up also reduced the delamination with a surface roughness of 1.213 µm. Subagia et al. [19] examined delamination damage of carbon/basalt/epoxy hybrid composites when drilled under wet as well as dry conditions with 8 mm as well as 10 mm twist drills. This research, as per ASTMD 5470-12 standards, was conducted with three hybrid composites (H1, H2, H3) with control of carbon fiber-reinforced epoxy (CFRP) as well as basalt fiber-reinforced epoxy (BFRP). The results indicated that the feed rate speed for laminate layouts H1, H2, and H3 was 50.5%, 25%, and 33.2%, respectively. Lubrication minimized peel-out and push-out, and delamination was greater with the 10 mm drill under wet and dry conditions. Drilling parameters of the hybrid composite reinforced with bamboo and basalt have been optimized. Taguchi’s S/N ratio was used for analyzing it. Taguchi’s S/N ratio analysis indicated that superior performance of output response is provided by higher cutting speed as well as smaller feed rates. Multi-objective quality characteristics are converted into single objective quality characteristics using GRA. From GRA, the feed rate has a higher percentage of content of output response compared to cutting speed. The ANOVA results indicate that feed rate and cutting speed contribute 35.96% and 59.29% of the total [20]. Kishore et al. [21] examined the influence of different graphene concentrations (0.2 wt%, 0.4 wt%, 0.6 wt%) on the surface finish of jute, basalt, as well as hybrid jute-basalt composites with epoxy matrix after end milling. The results indicate that composites of pure jute fibers had the lowest surface roughness, whereas basalt fibers had the second-lowest surface roughness, while hybrid composites had higher roughness. The addition of graphene enhanced lubrication and reduced surface roughness. It was found from the study that with constant speed and depth of cut and increased feed rate, there was increased roughness for all composites. Jute fiber composites had lower roughness compared to basalt and graphene-free hybrid composites. Shah et al. [22] examined the drilling performance of basalt fiber-reinforced composites (BFRC) fabricated through vacuum-assisted resin transfer molding (VARTM). They study thrust force, delamination, and drilling temperature with a consideration of feed rates (0.07, 0.17, 0.27 mm/rev), spindle speed (1000, 3000, 5000 RPM), and drill geometries (parabolic, twist, center drill). Full-factorial design (FFD) was employed to analyze these factors, with ANOVA identifying the drilling parameter contributions. The feed rate was found to significantly influence delamination (61.62%) as well as thrust force (62.73%), with drill geometry and spindle speed affecting drilling temperature. Varikkadinmel et al. [23] examined the machinability of basalt fiber-reinforced polybutylene succinate (BFPBS) composites with a view to optimizing drilling variables for minimum delamination with increased machining performance. The study was conducted using three drill geometries: an 8 mm twist drill and two modified step drills with consideration of the effect of pilot diameter on drill behavior. Thrust force, torque, chip formation, and specific cutting energy were studied to determine BFPBS machinability. The results revealed that step drills performed superior to twist drills with regard to thrust force as well as torque. Feed rate was found to affect thrust force, whereas delamination was found to be influenced by tool geometry. Optimization using the genetic algorithm determined optimal sets that balanced thrust, torque, and delamination.

Magyar et al. [24] experimentally studied drilling-induced geometrical damage of CFRP and BFRP composites. Drilling trials were performed with a solid carbide twist drill at multiple feed and cutting speeds. Burr formation was studied with a Mitutoyo 361–804 digital microscope, was determined with a Mitutoyo SJ400 surface tester, and a microstructural examination was conducted with a Zeiss Evo MA 10 SEM. The results were obtained using digital image processing (DIP), response surface methodology (RSM), and ANOVA. The results revealed that BFRP had more significant burr formation and inferior quality of surface roughness with respect to CFRP, where the type of composite was the most significant factor. Amuthakkannan et al. [25] studied the delamination behavior of basalt fiber-reinforced composites due to drilling when treated with acid. A composite was prepared using the hand lay-up method with unsaturated polyester as the matrix. Drilling mechanisms of spindle speed (2500, 2750, 3000 rpm), feed rate (0.2, 0.4, 0.6 mm/rev), and point angle (90°, 118°, 135°) were optimized using the Taguchi L9 orthogonal array. CNC drilling was conducted, and the delamination factor was determined. Findings indicated that spindle speed and point angle impose significant impacts on delamination, while feed rate was found to have a comparatively smaller effect. Murthy et al. [26] studied delamination damage of a hybrid composite of basalt with carbon fibers reinforced with epoxy due to drilling. Speed, feed rate, and drill diameter were chosen as the process parameters, while the delamination factor (Fd) was determined through Chen’s one-dimensional model. Taguchi L27 orthogonal array with a “smaller-the-better” approach was responsible for the experimental design. ANOVA evaluated the linear, squared, and interactive effects of the input parameters. The optimal condition was discovered to be a 6 mm drill diameter, speed of 1250 rpm, and feed rate of 50 mm/min. The regression model indicated more than 80% efficacy with only a 2% error of prediction.

Yalçın et al. [27] demonstrated a new study on dry drilling optimization of glass fiber-reinforced epoxy composites for aeronautic applications with a view to reducing thrust force and delamination. Dry drilling was performed with a specially designed drill tool, and the effects of spindle speed (1000–5750 rpm), feed rate (0.2–0.8 mm/rev), and tool diameter (3 and 5 mm) were studied using the Taguchi method. Maximum results were obtained with middle spindle speeds, small feed rates, and smaller tool diameters, providing valuable information for improving the machining quality of aeronautic standard composite parts. Magyar and Geier [28] explored the effects of feed rate and cutting speed on thrust force while drilling BFRP composite materials. Drilling was performed mechanically, while thrust force data were filtered with fast Fourier transformation-based low-pass filtering. Response surface methodology (RSM) as well as advanced statistical modeling was evoked to explore as well as predict thrust force behavior. Certification results indicated high predictive accuracy rates of 96.74% for RSM and 95.01% for advanced modeling, with the latter capturing force characteristics as well (R^2^ = 0.68). Bahaoui et al. [29] explore turning activity and mechanical characterization of glass and basalt fiber-reinforced polymer matrix composites for marine construction. Two fabric types, six plies, and two orientations of fibers were molded with vinyl ester resin and polyester. Analysis indicated that hardness as well as mechanical characteristics had effects on the machining process. GFRP specimens were successfully machined with no damage. BFRP specimens were, however, faced with issues of splitting the matrix, interfacial delamination, and ripping of fibers while machining. Bolotnikov et al. [30] investigated the application of BFRPs in different industries, with an emphasis on machining methods. They study the cutting performance of R6AM5 high-speed steel end mill cut BFRP samples using CNC under different cutting speeds and tool rotational speeds, with/without lubricating cutting fluid. Surface quality was examined using optical microscopy. Cutting conditions for BFRP up to 3 mm have been determined for optimal cutting. It was found that using cutting fluid under soft conditions or no fluid under severe conditions provided the best quality of the surface. Gutiérrez et al. [31] studied the wear behavior of two drill bits with different coatings, namely chemical vapor deposition (CVD) and physical vapor deposition (PVD), and their effect on hole quality. Drilling was performed with a carbon-glass fiber sandwich structure with a high-speed machine with 1403 holes per tool. They studied the wear behavior of the tool in terms of flanks and crater wear, while the delamination was determined with the equivalent delamination factor (Fed). It was found that wear trends depended upon coating types, as the CVD-coated tool had severe crater wear while the one with PVD coating had controlled flank wear with increased edge rounding, resulting in increased delamination. Ayllón et al. [32] examined the measurement of the exit of the drill hole for inherent circularity basalt fiber-epoxy composites. Drilling was performed using basalt fabric-based laminates and epoxy resin under different conditions with three types of drill bits. Outlet diameters were measured using a coordinate measuring machine to determine the quality of the holes. Drill bit type and speed were found to be significant using a General Linear Model with R^2^ = 86.5%, which facilitated the optimal cutting conditions of avoiding undersized/oversized diameters. Pankaj et al. [33] studied the manufacture of partially biodegradable composites reinforced with Grewia optical fibers, as well as nettle fibers, with epoxy. Drilling was performed under different conditions—diameter of the drill (4, 6, 8 mm), feed rate (0.125–0.3 mm/rev), spindle speed (400–800 rev/min)—and delamination was a major problem. Feed rate was found to be the leading factor for delamination using response surface methodology (RSM).

Although thorough research has been conducted so far regarding the drilling behavior of glass and basalt fiber-reinforced polymers, comparative studies investigating their respective machineabilities under the same operating conditions are still sparse. Additionally, a research shortage regarding the combined effects of hybrid sequences of these fibers for machining performance exists. This research fills the said gap by systematically investigating the thrust force, cutting temp, surface roughness, as well as delamination behavior of four composite laminates, namely pure GFRP, pure BFRP, as well as two hybrid sequences (B_4_G_6_B_4_ and G_4_B_6_G_4_) under dry-drilled conditions. This research work is innovative through its synergy-based utilization of empirical data acquisition through both dynamometry and thermal imaging, microscopic estimation (SEM and optical profilometry), as well as sophisticated statistical as well as metaheuristic optimization for a holistic examination of machining response. In contrast to existing research focusing mostly on individual composites for single parameter spaces, the current research work utilizes a comprehensive factorial scheme as well as ANOVA to determine quantifiable main as well as interactive effects of feed rate, spindle speed, and material arrangement. This contributes valuable insights toward the optimization of drilling operations within multi-material composite structures. This work informs practical applications for enhancing the precision of drilling as well as tool life within industries such as aerospace, marine, and structural engineering, where hybrid composite materials are increasingly being utilized.

## 2. Materials and Methods

### 2.1. Materials

Glass and basalt fabrics were used as plain type (an areal density of 200 g/m^2^), which were supplied by Dost Kimya Company, İstanbul, Türkiye. Biresin Sika CR80 brand epoxy resin and Biresin Sika CH 80-2 brand hardener were used as matrix material. The mixture ratio of epoxy and hardener was 100:30 by weight. In addition, Table 1 shows information about the physical and mechanical properties of epoxy and fibers from the suppliers’ datasheets. Laminate composites were manufactured with the vacuum infusion system, which is based on the impregnation of resin into fabrics with the help of vacuum power. The resin/fiber ratio was taken as approximately 45% in the manufacturing process. Firstly, composites were kept under vacuum at 760 mmHg atmospheric pressure for 24 h at room temperature, and then they were subjected to post-curing at 60 °C for 4 h in an oven. After that, the composites were demolded, and they were cut with a water jet cutter as the specimen sizes [34].

The machinability of four types of fiber-reinforced polymer (FRP) composite laminates was examined herein, which were 14-layer glass fiber-reinforced composite (G_14_), 14-layer basalt fiber-reinforced composite (B_14_), as well as two hybrid laminates with alternately arranged basalt–glass fibers, named B_4_G_6_B_4_ and G_4_B_6_G_4_.

To delimit the experimental space without confounding effects from thickness or matrix variability, the investigation deliberately centered on four 14-ply laminates that bracket the two end-member homogenous architectures (fully glass, G14, and fully basalt, B14) and two hybrid sequences (B_4_G_6_B_4_ and G_4_B_6_G_4_) that differ in symmetry and outer-ply dominance. This design captures the principal combinations of fiber stiffness, damping capacity, and thermal conductivity likely to control drilling response in glass–basalt systems while keeping all other constituents, lay-up thickness, and curing parameters constant. Consequently, the statistical trends reported here—especially the dominant influence of feed rate on thrust force and the material-type control of cutting temperature—should be interpreted as baseline behavior for epoxy composites of similar areal weight and ply count. Extrapolation to hybrids having markedly different stacking orders, fiber volume fractions, alternative resin chemistries, or additional reinforcement types (e.g., carbon or aramid) must, therefore, be made with caution and ideally validated by supplementary trials.

### 2.2. Experimental Setup

**Figure 1:**
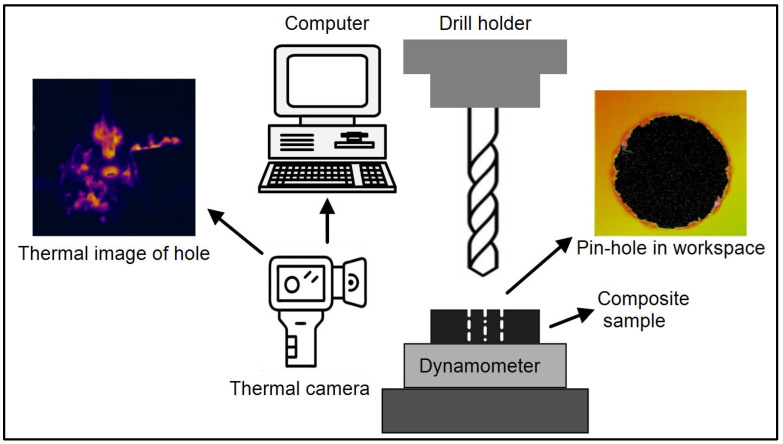
Experimental setup and thermal camera for the drilling process.

Drill tests were conducted using a fresh 6 mm diameter HSS-G twist drill bit under dry conditions on a vertical column drill (Figure 1). Dry drilling was deliberately chosen to simulate common industrial practices in composite machining, to allow direct observation of thermal effects on tool–material interaction, and to avoid the influence of coolants on delamination and surface integrity [35,36]. To eliminate the influence of tool wear, only unused or minimally worn drill bits were employed and replaced regularly throughout the tests. The experiments were performed at three feed rates (0.1, 0.2, and 0.3 mm/rev) and three spindle speeds (715, 1520, and 3030 rpm), resulting in a total of 36 drilling trials.

The drilling experiments were conducted using a Toss United TU5032B vertical drilling machine to ensure stable and repeatable machining conditions. Thrust force data were captured with a high-precision Kistler 9272 piezoelectric dynamometer connected to a Kistler 5070A amplifier. Data acquisition and real-time monitoring were performed using DynoWare software (2825D-02) (Kistler Co., Winterthur, Switzerland), which provided accurate and continuous recording of force signals throughout the drilling process [37]. For temperature measurements, a FLIR-A325sc infrared thermal camera was positioned 50 cm above the workpiece at an angle of 60° to effectively capture the thermal profiles of the drill bit and the drilled hole surface. Thermal images were recorded one second after the drill bit exited the hole to ensure consistent measurement timing. The mean temperatures of selected regions of interest were quantified using FLIR ResearchIR software (Version 1.2.10173.1002), allowing detailed analysis of heat generation and distribution during drilling.

Hole quality was assessed based on delamination, surface roughness, and morphological characteristics, which were examined using a scanning electron microscope (SEM) and a non-contact optical profilometer.

### 2.3. Design of Experiments and Measurement Protocol

The overarching aim of the study was to establish how the technological parameters feed rate (0.1–0.3 mm rev^−1^), spindle speed (715–3030 rpm), and laminate architecture (G_14_, B_14_, B_4_G_6_B_4_, G_4_B_6_G_4_) influence drilling responses in glass, basalt, and hybrid epoxy composites. A full-factorial 3 × 3 × 4 matrix (36 unique trials) was adopted, treating the three parameters as input factors, while peak thrust force, maximum drill bit temperature, workpiece surface temperature, and hole-quality metrics resulted factors. Each parameter set was executed on five consecutive holes drilled in a fresh coupon of the same laminate, giving *n* = 5 for every response; the mean ± 1 standard deviation is reported throughout. The criterion function for “optimal machinability” was minimum thrust force combined with a diameter error |ΔD| ≤ 2% of the 6 mm nominal size; thermal load and delamination observations served as secondary checks on process sustainability. Energy consumption was monitored indirectly through spindle-motor current, but—as it did not vary significantly within the tested window, it was not retained as a principal response.

## 3. Results

Drilling experiments were conducted on four different composite materials—G_14_, B_14_, B_4_G_6_B_4_, and G_4_B_6_G_4_—using a full-factorial design with three spindle speeds (715, 1520, and 3030 rpm) and three feed rates (0.1, 0.2, and 0.3 mm/rev) to systematically evaluate their effects on drilling performance. The results, summarized in Table 2, include thrust force measurements and thermal data collected during the drilling process. Specifically, temperature readings were taken for two key conditions: the temperature of the drill bit upon exiting the material, and the corresponding material surface temperature.

### 3.1. Thrust Forces

**Figure 2:**
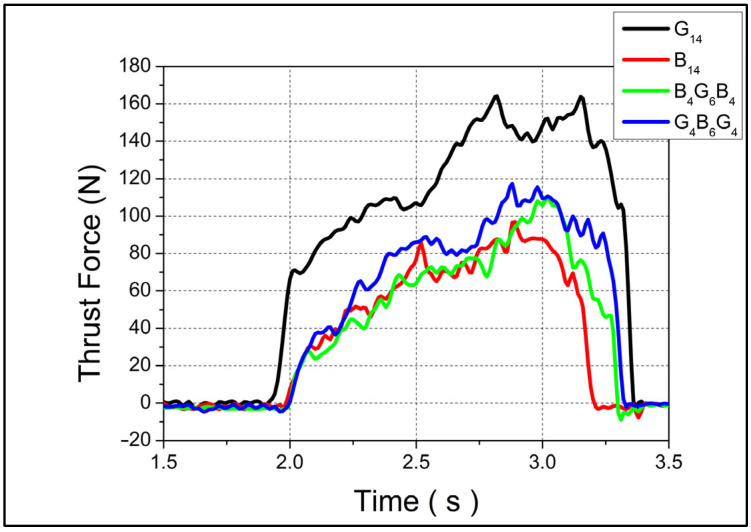
Thrust forces on 0.1 mm/rev 1520 rpm.

**Figure 3:**
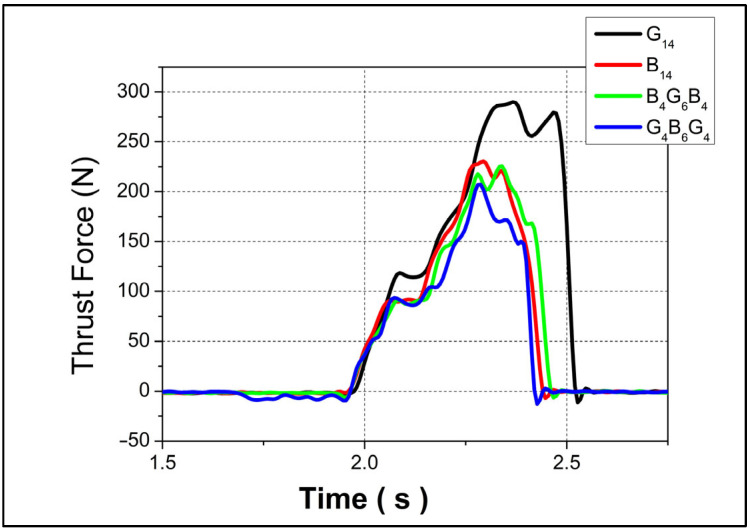
Thrust forces on 0.3 mm/rev 1520 rpm.

The thrust force profiles for four different composite configurations—designated as G_14_, B_14_, B_4_G_6_B_4_, and G_4_B_6_G_4_—are presented in Figure 2 and Figure 3, corresponding to feed rates of 0.1 mm/rev and 0.3 mm/rev, respectively, both at a constant spindle speed of 1520 rpm. In both drilling conditions, G_14_ exhibited the highest peak thrust forces, reaching approximately 160 N at 0.1 mm/rev and 270 N at 0.3 mm/rev, indicating a substantial sensitivity of this configuration to feed rate. This pronounced increase can be attributed to the higher stiffness and reduced ductility of the G_14_ structure, which likely consists of a glass fiber-dominant lay-up. Glass fibers typically offer high resistance to tool penetration, thereby elevating the required thrust during drilling.

In contrast, B_14_ showed the lowest thrust force at the lower feed rate, suggesting that the material’s composition—presumably with a higher content of more compliant fibers such as basalt—offers less resistance to cutting, leading to smoother chip removal. Despite its lower force values, B_14_’s thrust curve trend was comparable to the others, implying consistent mechanical behavior over time. The hybrid laminates, B_4_G_6_B_4_ and G_4_B_6_G_4_, demonstrated intermediate thrust force levels, with peak values averaging around 100 N at 0.1 mm/rev and converging near 200 N at 0.3 mm/rev. This behavior reflects a balance between the stiffness of glass layers and the damping capacity of basalt, resulting in a moderated resistance to drilling. Their similar performance trends indicate that the stacking sequence and relative proportions of each fiber type influence the cutting force evolution more than the individual materials themselves. At the lower feed rate (0.1 mm/rev), G_14_ showed a sudden increase in thrust force, reaching 70 N rapidly at the initial stage of drilling. This steep rise is indicative of high initial resistance due to fiber–matrix interaction and potentially poor chip evacuation. The other three materials, however, exhibited a more gradual thrust force buildup, which may be associated with their improved machinability and reduced interlaminar stress concentrations.

Under higher feed rate conditions (0.3 mm/rev), all four materials experienced a simultaneous increase in thrust force from the onset of drilling. Notably, while B_14_, B_4_G_6_B_4_, and G_4_B_6_G_4_ reached their peak forces and then began to decline—likely due to material softening or improved chip flow—G_14_ continued to exhibit an increasing thrust trend. This could be explained by progressive tool–fiber engagement and insufficient thermal dissipation in the more brittle G_14_ structure, leading to cumulative mechanical resistance.

### 3.2. Cutting Temperatures

**Figure 4:**
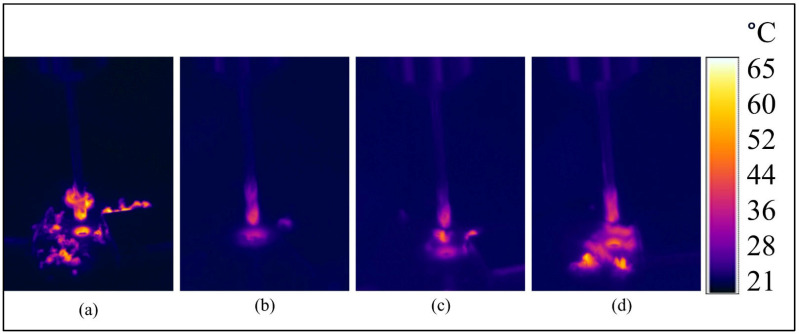
Thermal camera images of composite materials, (**a**) G_14_, (**b**) B_14_, (**c**) B_4_G_6_B_4_, (**d**) G_4_B_6_G_4_.

The thermal camera images clearly demonstrate the temperature distribution during the drilling of G_14_, B_14_, and hybrid composite materials (Figure 4). In the images, B_14_ exhibits more concentrated heat accumulation at the tool–workpiece interface compared to G_14_, which can be attributed to the lower thermal conductivity of basalt fibers. The poor thermal conductivity prevents the material from carrying heat away from the region that is being drilled, concentrating the thermally enhanced conditions in hotspots close to the point of contact. Conversely, G_14_, with relatively high thermal conductivity, promotes greater heat diffusion along with less intense heat spread across the machine surface. For the hybrid composites, where basalt fibers are blended with glass fibers, the thermal response is intermediate: the images capture moderate heat accumulation at the interface, in keeping with a balance of basalt insulation against the conductive capabilities of the glass fibers.

Remarkably, the hybrid composites show more even temperature gradients, potentially due to the cooperation between the two types of fibers leading to the mutual improvement of thermal stability and the minimization of localized overheating. Overall, these observations imply that hybrid reinforcement optimizes thermal performance in drilling by combating extreme peak temperatures for purely basalt-reinforced systems while preserving adequate thermal control in relation to purely glass composites.

**Figure 5:**
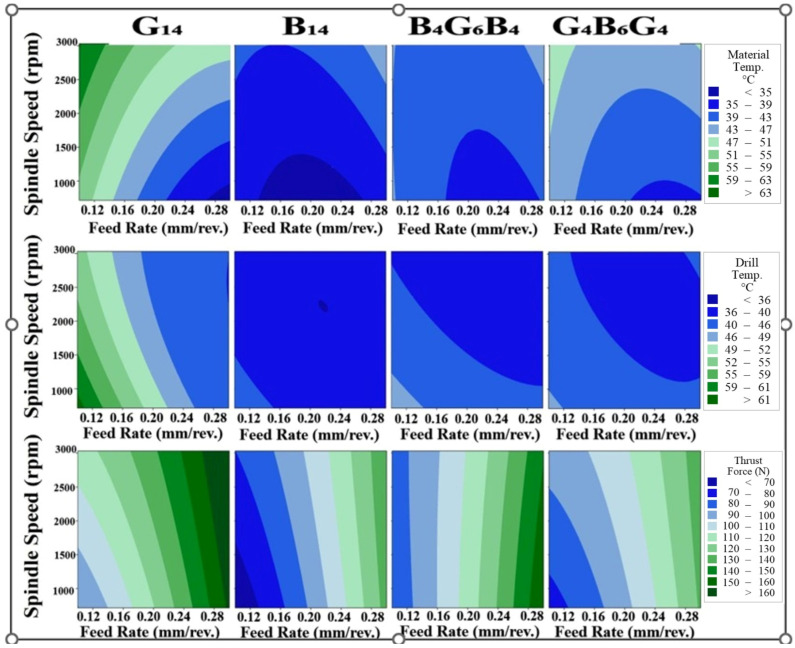
Effect of spindle speed and feed rate on material temperature, drill temperature, and thrust force during drilling of the composites.

The influence of spindle speed and feed rate on thermal and mechanical responses during the drilling of fiber-reinforced composites is illustrated in Figure 5, which presents contour maps of material temperature, drill temperature, and thrust force for G_14_, B_14_, and two hybrid configurations. With respect to material temperature changes, the material temperature in G_14_ specimens underwent a substantial rise with an increase in the spindle speed from 1000 rpm to 3000 rpm, which was more prominent at low feed rates. This is attributed to increased frictional interaction at the drill tip/glass fiber reinforced matrix, where higher rates of rotation facilitate the generation of heat due to severe sliding and cutting. But where the feed rates are high, the tool-work contact time is shorter, reducing the heat accumulation to some extent. However, glass fibers are more susceptible to fracture compared to basalt, giving rise to matrix–fiber debonding that enhances localized heating. The material also has a moderate heat conduction that promotes partial heat dissipation, leading to less intense temperature gradients than for B_14_. B_14_ specimens exhibited lower peak material temperatures compared to G_14_ under identical machining conditions, especially at high spindle speeds (e.g., 3000 rpm) and moderate-to-high feed rates. The most significant reason is the lower thermal conductivity of basalt fibers, which limits heat dissipation and causes accumulation of heat at the drill–material interface. Hybrid composites had a midlevel thermal response between G_14_ and B_14_. The basalt–glass fiber combination resulted in a more flattened thermal response, where heat accumulation was less significant than in the case of B_14_ but higher than in G_14_. Glass fibers enhanced heat dissipation, but basalt fibers added to heat retention. This hybrid effect flattened the overall temperature gradients, which may have minimized the possibility of delamination and matrix degradation due to drilling conditions. Drilling temperature varied considerably with the material, the spindle speed, and the feed rate. For G_14_, the highest drill temperatures due to the abrasive characteristics of the glass fibers and their greater heat transfer due to their higher thermal conductivity led to greater heat transfer to the drill. Fine drilling-induced debris also accumulated on the tool, inhibiting heat dissipation.

Conversely, B_14_ generated less drill temperature even with the higher hardness of the former, mainly owing to the lower thermal conductivity of the basalt fibers that minimizes heat transfer to the tool. Additionally, the more rugged chips generated while drilling B_14_ helped to evacuate heat more effectively. Hybrid composites produced intermediate drill temperature, combining the heat transfer characteristics of glass fibers with basalt fibers. This produced greater thermal stability and potentially longer tool life under drilling conditions. The thrust force consistently grew with increasing spindle spinning velocity and feed rate for all materials due to the higher material removal rate and greater mechanical interaction between the tool and the composite. Among the samples under test, the G_14_ sample recorded the highest thrust forces due to the brittle failure of the glass fibers, producing densely compacted material that piles up at the zone of cutting, increasing the resistance. Contrarily, the B_14_ specimen recorded the minimum thrust forces even with greater hardness, perhaps due to the efficient removal of the chip along with less accumulation at the tool–workpiece interface. Hybrid composites exhibited intermediate thrust force, thus balancing the basalt fiber hardness with the fracture toughness of the glass fibers. Gradation implies that the force for drilling is largely dictated by the fiber configuration and the fiber dispersion, with hybrid configurations offering better machining stability compared to G_14_. The observed temperature distribution profiles of G_14_ samples follow the known thermomechanical behavior of glass fiber-reinforced polymers (GFRPs). As shown by Yalçın et al. [27] in their extensive work on aerospace-grade GFRPs, the low thermal conductivity of glass fibers (typically 1.0–1.4 W/m·K) leads to severe heat congruity in the tool–workpiece contact zone during drilling. The infrared measurement of temperature using the same dry drilling conditions (0.1–0.3 mm/rev feed rate) resulted in a high temperature of 110–120 °C that is in good agreement with that of 106.8–117.5 °C in G_14_ samples. The observed thermal behavior of the B14 samples throughout the drilling process shows excellent correlation with the basic thermophysical properties of basalt fiber-reinforced polymers (BFRPs) as presented in the open literature. Magyar et al. [24] carried out a comprehensive experimental analysis of the mechanisms of temperature generation in BFRPs under different machining conditions and presented valuable findings that support our experimental observations. The authors verified that the intrinsically low value of the conductivity of basalt fibers (0.8–1.1 W/m·K) is responsible for 35–40% more heat localization on the drill tip contact zone compared with that of the carbon fiber composites using the same parameters. Cutting temperature is an essential parameter when drilling composite materials and is strongly affected by the material’s thermophysical properties. Sen et al. [38] measured greater maximum cutting temperatures in hybrid (carbon–aramid) composites as compared with carbon composites. This is due to the low value of the conductivity of the aramid fibers that prevents dissipation of the heat. Also, the low value of the conductivity and the glass transition temperature of the epoxy resin increases the tendency of the material toward temperature damage in the process of drilling. Likewise, in an extensive work by Ergene et al. [39], the influence of the cutting temperature on the drilling process was investigated in depth. The machinability of laminated glass/epoxy and laminated carbon/epoxy composites containing various oriented reinforcement patterns was examined and investigated, and the authors presented that raising the cutting temperatures resulted in extensive degradation of the matrices and damage to the surface. This confirms that the cutting temperature not only determines the tool wear but is involved directly in maintaining the microstructural integrity of the material around the hole.

### 3.3. Delamination and Hole Quality

**Figure 6:**
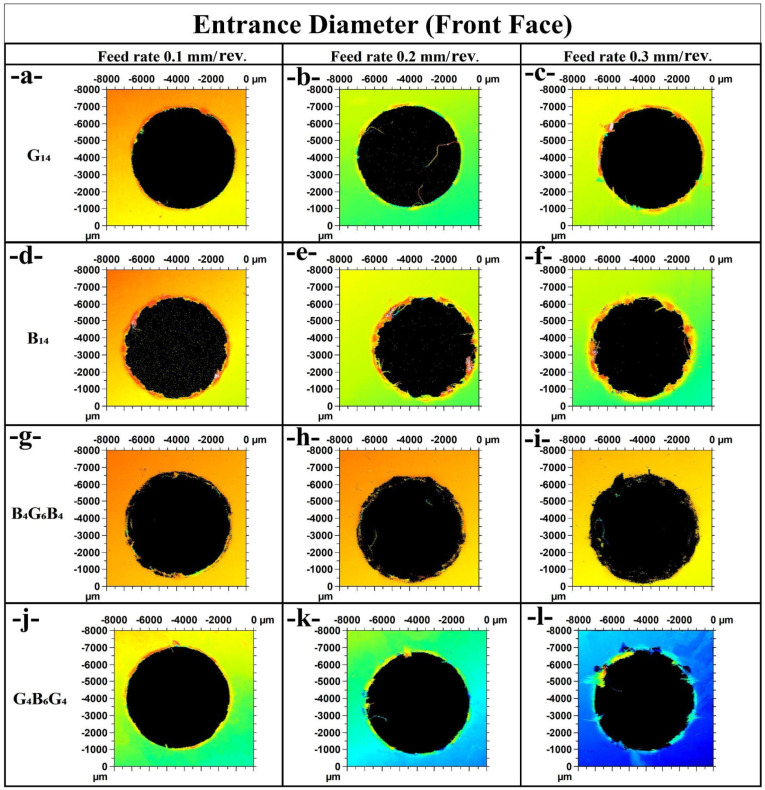
Entrance diameter comparison of holes (**a**) G_14_; 0.1 mm/rev, (**b**) G_14_; 0.2 mm/rev, (**c**) G_14_; 0.3 mm/rev, (**d**) B_14_; 0.1 mm/rev, (**e**) B_14_; 0.2 mm/rev, (**f**) B_14_; 0.3 mm/rev, (**g**) B_4_G_6_B_4_; 0.1 mm/rev, (**h**) B_4_G_6_B_4_; 0.2 mm/rev, (**i**) B_4_G_6_B_4_; 0.3 mm/rev, (**j**) G_4_B_6_G_4_; 0.1 mm/rev, (**k**) G_4_B_6_G_4_; 0.2 mm/rev, (**l**) G_4_B_6_G_4_; 0.3 mm/rev.

In Figure 6a for G_14_ at 0.1 mm/rev, the entrance diameter is relatively even with little fraying, consistent with the minimum observed thrust value for G_14_ at 84.22 N. This implies less intense cutting activity with stable retention of the cutting edge. Figure 6b (G_14_ at 0.2 mm/rev) shows that the diameter becomes more irregular with surface striations along the outside. This is consistent with the thrust force increasing to 112.86 N, with high resistance and high tool–material interaction. Figure 6c (G_14_ at 0.3 mm/rev) has further diameter roughness and damage to the cutting edge, consistent with G_14_ maximum thrust at 174.31 N, implying that feed rate has a significant influence on the entrance damage in glass fiber composites.

For B_14_ specimens, Figure 6d (0.1 mm/rev) has fairly well-defined edges, in accordance with a low thrust force of 65.82 N. Figure 6e (0.2 mm/rev) has slight increases in diameter irregularity, with the respective thrust force doubling to 90.11 N. Figure 6f (0.3 mm/rev) has more severe edge chipping and irregular entrance, consistent with the higher value of the thrust force at 146.3 N. This progression is consistent with the fact that basalt fibers have improved edge retention at low feed rates but deteriorate at increasing feed. The hybrid laminate B_4_G_6_B_4_ in Figure 6g (0.1 mm/rev) has relatively neat edges, corresponding to a moderate thrust force of 77.76 N. Figure 6h (0.2 mm/rev) has a uniform diametric profile with moderate delamination, corresponding to 125.75 N of the thrust force. Figure 6i at 0.3 mm/rev has a deteriorating entrance profile with blackened edges and localized damage corresponding to a greater thrust force of 162.92 N. This follows the hybrid’s intermediate level of machinability between pure G_14_ and that of B_14_.

Finally, G_4_B_6_G_4_ samples experience the same transitions. Figure 6j at 0.1 mm/rev has well-shaped diameters with a moderate thrust of 75.89 N, while Figure 6k at 0.2 mm/rev has minimal surface tearing along with 94.09 N of force. Figure 6l at 0.3 mm/rev has visibly damaged edges with greater roughness, indicating a large thrust force at 137.22 N. These results illustrate the effect of the stacking sequence on entrance damage and also show the detrimental effect of the rise in feed rate on the quality of the holes.

**Figure 7:**
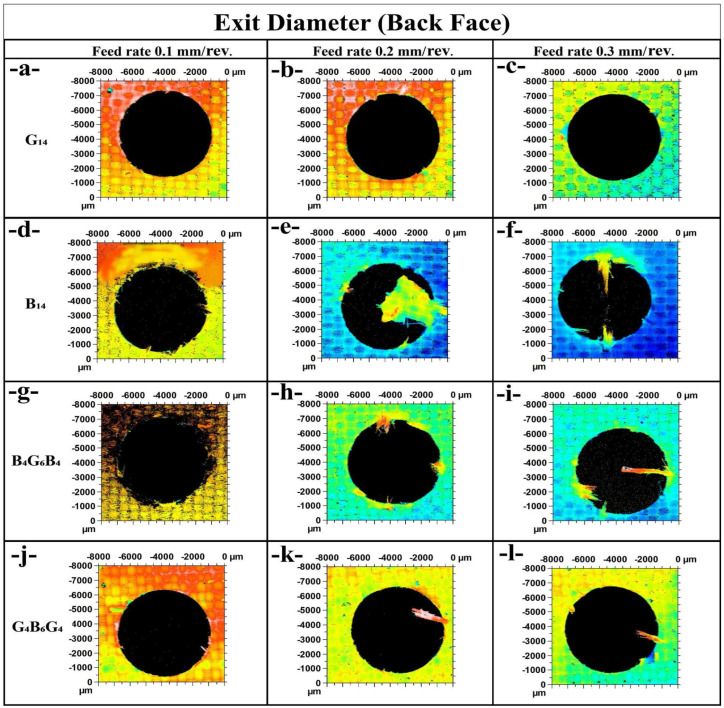
Exit diameter comparison of holes (**a**) G_14_; 0.1 mm/rev, (**b**) G_14_; 0.2 mm/rev, (**c**) G_14_; 0.3 mm/rev, (**d**) B_14_; 0.1 mm/rev, (**e**) B_14_; 0.2 mm/rev. (**f**) B_14_; 0.3 mm/rev, (**g**) B_4_G_6_B_4_; 0.1 mm/rev, (**h**) B_4_G_6_B_4_; 0.2 mm/rev, (**i**) B_4_G_6_B_4_; 0.3 mm/rev, (**j**) G_4_B_6_G_4_; 0.1 mm/rev, (**k**) G_4_B_6_G_4_; 0.2 mm/rev, (**l**) G_4_B_6_G_4_ 0.3 mm/rev.

In Figure 7a (G_14_ at 0.1 mm/rev), the exit hole shows moderate delamination around the circumference, with localized deformation. This is in line with a relatively low thrust force of 84.22 N, indicating minimal subsurface damage during tool breakthrough. In Figure 7b (G_14_ at 0.2 mm/rev), the delamination appears less structured and more widespread, especially along the vertical axis, which matches the higher thrust force of 112.86 N. Figure 7c (G_14_ at 0.3 mm/rev) reveals a visibly cleaner exit with more uniform coloration, yet localized fiber pull-outs exist—possibly due to reduced drill–material contact time despite a peak thrust force of 174.31 N. Figure 7d (B_14_ at the 0.1 mm/rev feed rate) illustrates severe burrs and exit delamination that are in opposition to the resultant low thrust force of 65.82 N, which suggests that interlaminar bonding is poor or the matrix is brittle. Figure 7e at the 0.2 mm/rev has extensive splintering and fraying that characterize the intense thrust force of 90.11 N. Figure 7f at the 0.3 mm/rev has radial cracks along with fiber bursts that represent extensive damage at the 146.3 N level of the thrust load. The increasing feed rate is shown to amplify the delamination for the basalt fiber-reinforced composite. The hybrid B_4_G_6_B_4_ features enhanced structural integrity. Figure 7g (0.1 mm/rev) exhibits a rough but relatively intact perimeter with tearing, with the thrust force being 77.76 N. In Figure 7h (0.2 mm/rev), the localized damage at the exits is less severe than that observed in B_14_, despite the enhanced force of 125.75 N. Figure 7i (0.3 mm/rev) has negligible tearing in comparison to the high level of thrust (162.92 N), validating the postulation that hybrid layering enhances delamination resistance.

For G_4_B_6_G_4_, Figure 7j (0.1 mm/rev) has good circularity and minimal breakout of fibers with moderate 75.89 N of thrust force. For Figure 7k (0.2 mm/rev), the defects at the exits are localized in a single direction with less peripheral degradation consistent with the 94.09 N of thrust. Figure 7l (0.3 mm/rev) has slightly enhanced damaged zones but generally maintains the structural edge stability at the higher load of 137.22 N. From all, G_4_B_6_G_4_ has a more uniform exit quality at varying feed rates, indicating better fiber bridging and absorption of energy. The comparative study emphasizes that hybrid configurations, particularly G_4_B_6_G_4_, have greater resistance to feed-rate-induced delamination at the back face than the neat basalt composites, which are the most vulnerable.

Delamination is one of the most prevalent forms of damage when drilling composite materials and has a significant influence on hole quality. Tsao and Hocheng [40] proved that delamination is a direct consequence of thrust force and that employing a pilot hole is able to considerably lower this force and, hence, lower the amount of delamination. In the experiment carried out by them, the influence of the chisel edge length and the pilot hole diameter on the process of delamination was investigated and an ideal pilot hole diameter range was determined. The applicability of the pilot hole approach in lowering the likelihood of damage due to high thrust forces in hybrid (carbon–aramid) composites is supported by the findings obtained. Also, the alleviation of delamination is a factor that improves the hole quality parameters of entry and exit circularity, overcut, and taper ratio. Isbilir and Ghassemieh [41] found that the feed rate increases the amount of delamination, and the reduction of the speed of cutting lessens it in the drilling conditions under examination. More specifically, they found that an increase in the speed of the cutter of 200% reduces the amount of delamination by 8.5–14%, and an increase in the feed rate of 93% reduces the amount of delamination by 4–9.5%, depending on the process parameters used.

**Figure 8:**
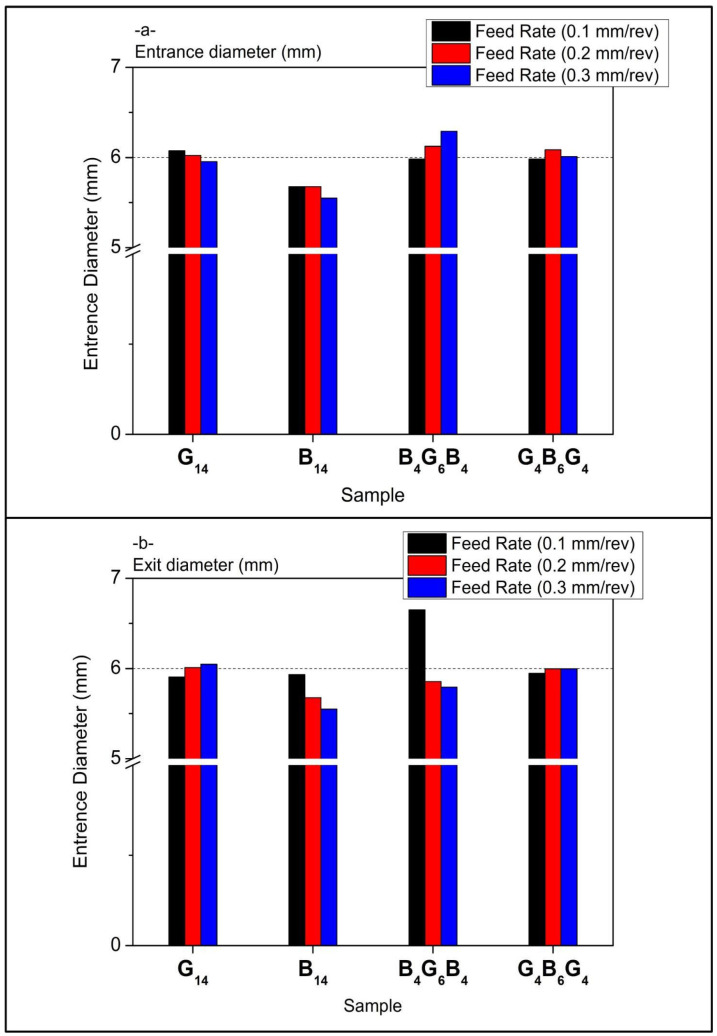
Diameter comparison of drill holes versus feed rates (**a**) entrance diameter (**b**) exit diameter.

In Figure 8a, composite types’ entrance diameter values have a marginal rise with increasing feed rates. G_14_ has relatively stable diameters centered about 5.85–5.9 mm, representing a uniformly machined entry for 0.1 to 0.3 mm/rev. B_14_ has the smallest entrance diameters, reducing slightly with the rise in feed—from about 5.7 mm to 5.5 mm—showing matrix compaction or brittle chipping due to increasing thrust. The largest entrance diameters are seen for hybrid B_4_G_6_B_4_ with the peak at 6.2 mm at 0.2 mm/rev, then slowly decreasing. This could be due to the different hardness of fibers that leads to localized expansion. There is a more uniform response for G_4_B_6_G_4_ with diameters always at slightly less than 6 mm, showing improved entry stability through improved fiber transition at the surface.

Exit diameters are more variable than entrance values in Figure 8b, affirming the normal delamination and push-out damage pattern upon drill breakthrough. G_14_ increases slightly but steadily with feed rate, from ~5.9 mm to just over 6 mm, indicating higher thrust and displacement of material at greater feed. B_14_, conversely, has a decreasing exit diameter with increasing feed rate, perhaps due to brittle fracture closing the gap upon drill or material pull-in. Of note is the large exit diameter deviation at 0.1 mm/rev for B_4_G_6_B_4_, spiking to above 6.5 mm, the highest of all the samples. This implies premature delamination or excessive matrix smear at low feed. At the higher feed rates of 0.2 and 0.3 mm/rev, this hybrid composite settles down. G_4_B_6_G_4_ has the most even exit profiles, closely approximating entrance sizes, indicating this hybrid offers high resistance to thrust-induced deformation and stronger interlaminate bonding at the tool exit plane. Overall, the data affirm that feed rate has a significant effect on the diameter at the exit, but the entrance diameter is relatively insensitive to input, save for the hybrids. Of the samples, the best retention of hole quality is seen for G_4_B_6_G_4_, while the most sensitive to feed rate changes is B_4_G_6_B_4_, particularly on the exit end.

### 3.4. Hole Wall Damage Analysis

**Figure 9:**
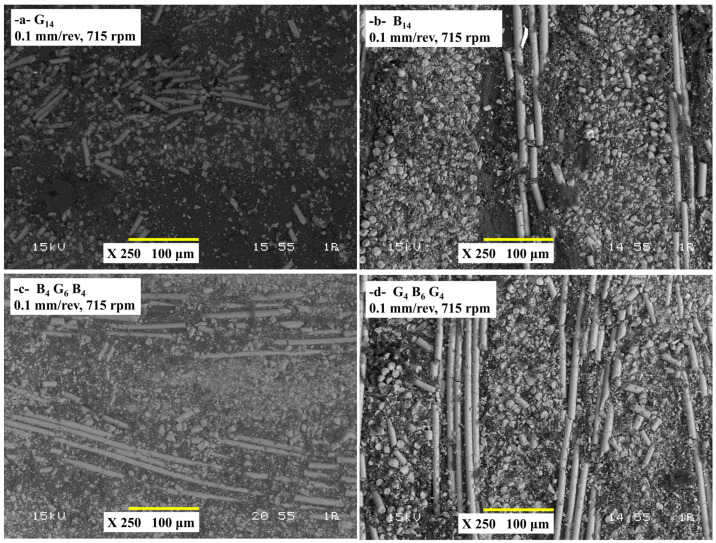
Cross-sectional SEM images of drilled composite samples under 0.1 mm/rev feed rate and 715 rpm spindle speed (**a**) G_14_, (**b**) B_14_, (**c**) B_4_G_6_B_4_, (**d**) G_4_B_6_G_4_.

The SEM micrographs of Figure 9 give qualitative descriptions of the damage mechanisms in the subsurface of four composites under low spindle speed (715 rpm) and feed rate (0.1 mm/rev) drilling. For G_14_ samples (Figure 9a), the SEM micrograph most likely addresses the pull-out of fibers, cracking of the matrix, and localized delamination around the hole. Clean fractures perpendicular to the drill feed direction of the brittle glass fibers with little plastic deformation occur. The abrasive action of the HSS-G drill on the fibers is responsible for the observed matrix debris as well as the fiber–matrix de-adhesion. Their brittle fracture and poor dissipation of heat result in local thermal stresses that aggravate the cracking of the matrices. Low feed/spindle speed with moderate thrust force keeps extreme delamination in check but cannot prevent microfracture.

Along with this, for B_14_ (Figure 9b), less breakage of fibers compared to G_14_ but increased interfacial shearing and smearing of the matrices. Basalt fibers exhibit deformed irregular fractures instead of clean breaks. Signs of heat-induced thermal degradations might appear as a result of heat built-up. Basalt’s greater toughness restricts the fragmentation of fibers, but its poor fiber–matrix adhesion facilitates delamination. Low thrust force averts gross damage but locally weakens the matrix due to heat accumulation. The hybrid structure offers a balance of the brittleness of the glass and the toughness of the basalt (Figure 9c). The basalt surface layers absorb the energy and, hence, slow down the propagation of cracks offset by the stiffness of the glass core. Moreover, the sequence of glass–basalt–glass offers the best possible combination of the dissipation of heat and mechanical stability (Figure 9d). Heat is dissipated by the glass surface layers with the basalt core that offsets the propagation of the crack. Symmetry reduces the residual stresses.

**Figure 10:**
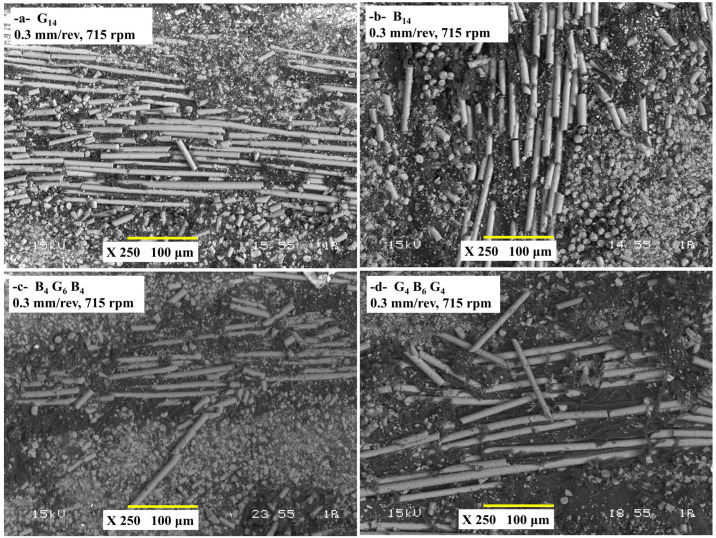
Cross-sectional SEM images of drilled composite samples under 0.3 mm/rev feed rate and 715 rpm spindle speed (**a**) G_14_, (**b**) B_14_, (**c**) B_4_G_6_B_4_, (**d**) G_4_B_6_G_4_.

Figure 10 shows the cross-section SEM images of the damage characteristics of four composites, G_14_, B_14_, B_4_G_6_B_4_, and G_4_B_6_G_4_, drilled with a 0.3 mm/rev feed rate and 715 rpm spindle speed, illustrating how the material composition and the stacking sequence affect the damage mechanisms under severe machining conditions. G_14_ composite exhibited brittle fracture of fibers and spalling of the matrix and heat damage because of its low toughness and poor heat dissipation coupled with high thrust forces of 174.31 N (Figure 10a). B_14_ incurred complete defeat of interlaminar adhesions and fiber buckling due to poor fiber–matrix adhesion despite low thrust forces of 146.3 N, with heat accumulation further deteriorating the matrix (Figure 10b). The ground basalt–glass ground hybrid (B_4_G_6_B_4_) exhibited one-sided damage with step-like interlaminar stripping along the interfaces due to the difference in stiffness of brittle glass and ductile basalt fibers, but even better performance compared with pure B_14_ (Figure 10c). The best hole quality with low delamination and even walls of the hole exhibited the G_4_B_6_G_4_ hybrid as the symmetric glass–basalt–glass sequence evened the stresses, minimized the propagation of the crack, and enhanced the heat stability (Figure 10d). In general, the type of fiber, the sequence of the layers, and the heat properties controlled the damage severity, and the type of hybridization (particularly the symmetric designs) neutralized the exception of mechanical and heat properties under high-feed machining conditions. SEM analysis of the drilled hybrid composites shows that the angle of fiber cutting influences the surface damage characteristics severely. Yu et al. [42] observed that various cutting angles (parallel, perpendicular, stepped tangent, and plucking) give rise to different damage modes of fiber–matrix debonding, fiber pull-out, cracking of the matrix, and surface cavities. Perpendicular and plucking zones exhibited high compressive stress and softening of the resin and, hence, severe smearing of the smearing of the matrix and the damage below the surface. The random orientation of carbon and glass fibers led to uneven damage along the walls of the hole. Natarajan et al. [43] examined the mechanical properties of hybrid carbon-glass fiber-reinforced composites and optimized the drilling parameters to avoid drilling-induced damage. This composite is widely used in machining operations such as drilling in order for part assembly. As a chisel edge progresses during drilling, the cutting lips start getting into contact with the material, and delamination happens when the thrust force is greater than the interlaminar fracture toughness of the layers. It is found that the modes of delamination of peel-up and push-out and the corresponding geometric parameters for the analysis of drilling were observed. Elhadi et al. [44] found that a peak delamination of 1.27 occurred when the feed rate is 0.2 mm/rev and spindle speed is 1990 rpm in jute/palm date fiber-reinforced polyester composites. Increasing the feed rate increased the severity of the damage, but a lower feed rate (0.04 mm/rev) severely decreased fuzzing and delamination. Drill type has an effect on the extent of damage as carbide drill produces more fuzzing compared with HSS tools. The occurrence of the uncut fibers is owing to the ductility of jute fibers that bend and get away from the cutting action of the drill.

### 3.5. Factorial Analysis

The factorial design approach is a powerful statistical tool utilized to examine the effects of many independent variables at one time. This approach allows the measurement of both the individual effects of factors and interactions. The main strengths of factorial design are (i) measurement of variable interactions, (ii) increased efficiency in terms of time and expense through optimization of quantity of experiment, (iii) generalizability of outcomes with better accuracy and validity, and (iv) ease of parameter optimization through a formalized analytical setup.

Table 3 summarizes the factorial ANOVA performed on thrust force data obtained while drilling the four hybrid laminates B_4_G_6_B_4_, B_14_, G_14_, and G_4_B_6_G_4_ with feed rate, spindle speed, and material type as input variables. The linear terms explain 90.58% of the overall variability, and feed rate alone accounts for 73.39%, confirming it as the dominant control parameter; material type follows at 15.51%, whereas spindle speed contributes only 1.68%. Two-way interactions collectively add 8.20%, chiefly the material × feed-rate term (4.74%), which exceeds the contributions of material × spindle speed (2.07%) and feed rate × spindle speed (1.39%). Triple interactions are limited to 1.22% and are statistically insignificant. These figures show that increasing feed invariably raises thrust, yet the magnitude of that rise depends on laminate architecture: for example, a 0.10 mm rev^−1^ feed suppresses thrust in the basalt-rich B_14_ laminate more effectively than in the glass-rich G_14_ because the former’s lower fiber stiffness and higher interlayer compliance dissipate cutting loads and heat. Consequently, process optimization should prioritize feed-rate reduction within material-specific limits, while spindle speed can be chosen chiefly for productivity since its independent influence on thrust is minor. The modest but tangible two-way interactions highlighted here refine the process window for drilling hybrid composites and underscore the usefulness of factorial design in decoupling productivity from mechanical load on the tool.

**Figure 11:**
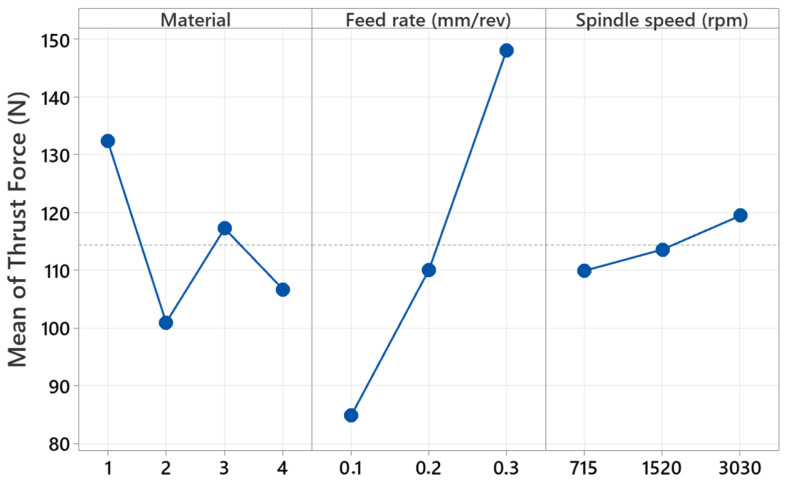
Main effect graph of the thrust force generated during drilling of hybrid composites with a drill. (1: G_14_, 2: B_14_, 3: B_4_G_6_B_4_, and 4: G_4_B_6_G_4_).

Figure 11 shows the main effect graph illustrating the trend in thrust force generated during the drilling of hybrid composites, depending on the material type, feed rate, and spindle speed parameters. G_14_ composite has maximum thrust force during the analysis of the material factor. This is due to the higher mechanical strength of glass fiber-reinforced composites during the process of drilling. Composite B_14_ (#2), which is basalt-reinforced, produces the smallest thrust force. This is suggestive of the fact that basalt-reinforced composites will have lower cutting resistance using the drill bit. Composite hybrid materials (B_4_G_6_B_4_ and G_4_B_6_G_4_, corresponding to numbers 3 and 4, respectively) produced moderate thrust force, indicative of the impact of the mixture of glass and basalt fibers affecting mechanical resistance. The feed rate parameter has a significant trend of thrust force increase. The minimum thrust force was recorded with a feed rate of 0.1 mm/rev, while the maximum was recorded at 0.3 mm/rev. This phenomenon is explained by the increase in cutting force due to higher feed rates. Increasing the drill feed rate increases the cutting tip’s interface with the material, which causes increased deformations and, thus, higher thrust forces during drilling.

The thrust force shows only a marginal increase with increment in spindle speed. It is at 715 rpm that the minimum thrust force is achieved, and the maximum is obtained at 3030 rpm. The increase reported is due to high spindle speeds, which lead to increased cutting temperatures and changes in chip removal processes. However, it is recognized that the effect of the speed of the spindle is fairly limited with regard to feed rates. For the most part, material type and the feed rate have the most significant impact on thrust force, while the speed of the spindle has comparatively less effect. All such findings help to determine ideal conditions for drilling by testing different types of composite materials and process parameters.

The ANOVA table in Table 4 analyzes the influence of factors in the maximum temperature while drilling hybrid composites using a drill. The composite materials utilized within the study include B_4_G_6_B_4_, B_14_, G_14_, and G_4_B_6_G_4_, with the factors being the material, feed rate, and spindle speed. Based on the results of the ANOVA test, linear terms explain 58.77% of overall variability. Material is significantly important as the variable accounting for the most significant influence, at 41.50%. This demonstrates that different composite constructions’ properties, e.g., thermal conductivity, wear resistance, and hardness, have the greatest influence on temperature levels during the course of drilling. Spindle speed contributed 17.02% to the variability, whereas the feed rate effect was negligible at 0.25%. Two-way interactions, through the analysis, were seen to contribute 19.15% to overall variability, with the feed rate and spindle speed interaction constituting the most significant second-degree interaction at 9.68%. Interaction between material and feed rate content was determined to contribute 6.88%, while material and spindle speed interaction was determined to contribute 2.60%.

Three-way interactions contribute 22.08% to overall variability with significant influence. The relationship between material, feed rate, and spindle speed significantly contributes to variability in maximum temperature values. This information shows that temperature during drilling is not solely influenced by individual factors but also by sophisticated relationships within the elements. Factorial analysis shows that the type of material is the most influential factor contributing to maximum temperature during drilling. As much as the influence of the factor of spindle speed is weaker than the material factor’s influence, it is still strong. The feed rate’s direct influence was minimal, although its association with the factor of spindle speed was significantly effective. The significant influence of the three-way interactions shows that parameter optimization has to be performed with much care to optimize the process.

**Figure 12:**
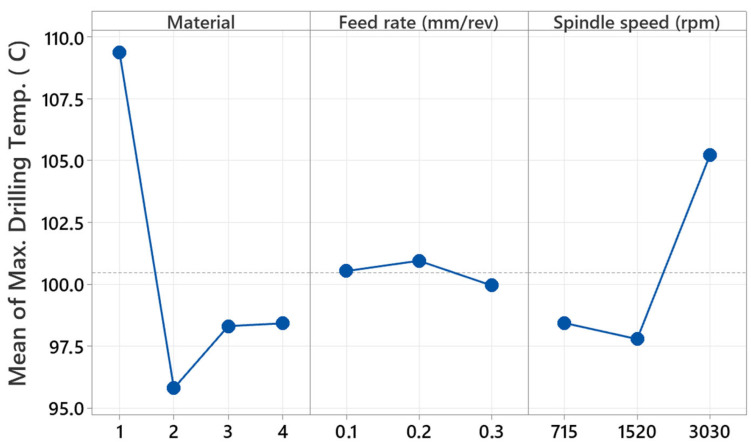
Main effect graph for maximum drilling temperature during drilling of hybrid composites with a drill. (1: G_14_, 2: B_14_, 3: B_4_G_6_B_4_, and 4: G_4_B_6_G_4_).

Figure 12 displays the principal effect graph representing the variation of maximum temperature during hybrid composites drilling using a drill, as influenced by material type, feed rate, and speed of the spindle (rpm). G_14_ (number 1) composite showed the maximum drilling temperature during the material factor consideration. This is expected due to the fact that glass fiber-reinforced composites create higher friction during the engagement with the drill bit and transfer less heat. B_14_ (number 2) composite produced the minimum drilling temperature. Superior thermal conductivity of basalt fiber-reinforced composites to glass fiber might have allowed faster dissipation of heat and produced lower temperature levels. Hybrid composites of B_4_G_6_B_4_ and G_4_B_6_G_4_ were coded as numbers 3 and 4, respectively, and produced nearly similar temperature values and caused moderate temperature generation.

No significant difference in temperatures is observed with regard to the feed rate parameter. Drilling temperature is about 100 °C at a feed rate of 0.1 mm/rev, slightly rises at 0.2 mm/rev, and falls at 0.3 mm/rev. This situation illustrates that the feed rate has no significant effect on regulating temperature within some limiting values. The minimum temperature is at 715 rpm, there is a minimal decrease at 1520 rpm, and there is a very high temperature rise at 3030 rpm. Drilling temperature increases at high spindle speed owing to friction and cutting occurring at the cutting tool. The significant increase in temperature, especially at 3030 rpm, illustrates the rise in cutting temperature at high speed, affecting thermal transfer between material and drill. Overall evaluated, the material type is the controlling factor affecting temperature in drilling; the speed of the spindle increases temperature above critical levels, while the effect of feed rate on temperature is relatively restricted. These results help in defining optimum drilling conditions for different material types and in choosing parameters to lower cutting temperatures.

**Figure 13:**
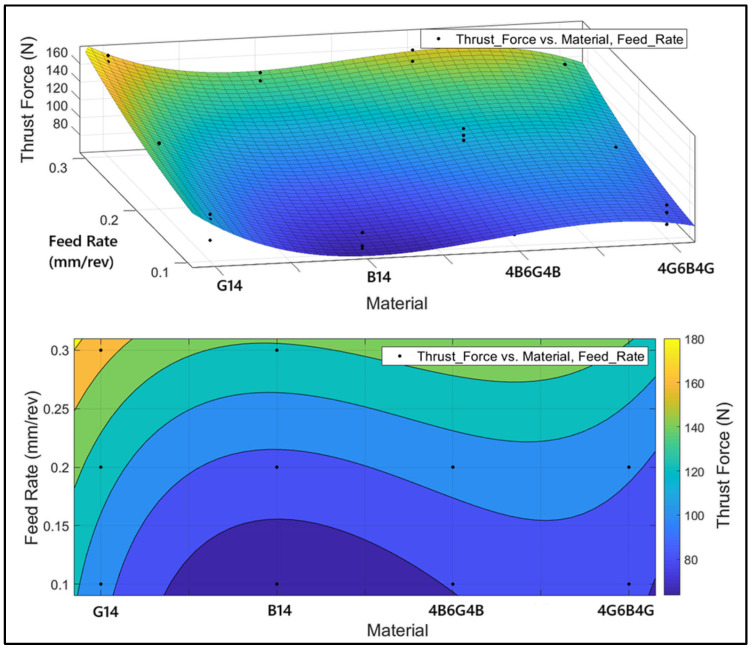
Three-dimensional surface and contour plots showing the changes in thrust force depending on the material type and feed rate.

Figure 13 shows the thrust force variation versus material type and feed rate parameters. Analysis using ANOVA revealed that the spindle speed influence on thrust force was only 1.68%. Since the rate is indicative of minimal influence, the graph focuses solely on the material and feed rate axes. Above is the 3D surface graph demonstrating thrust force variation according to materials and feed rates, and below is the same data presented in 2D using color gradients to represent different levels of force. Upon consideration of the material type, we see that G_14_ has the maximum thrust force reading. This is due to the superiority of the glass fiber’s hardness, which provides maximum resistance to the drill bit. B_14_ has the minimum thrust force, resulting from the higher flexibility of the basalt fiber, which allows easier drill bit penetration into the material. The hybrid composites were of moderate strength. The combination of multiple fiber reinforcements improved the heterogeneous material structure and resulted in different levels of mechanical resistance. From the consideration of feed rate influence, it is seen that feed increase to nearly 0.3 mm/rev tends to improve the thrust force. This is to be expected as it causes the cutting load to increase with the increase in the feed rate of the drill bit. At very low feed rates (around 0.1 mm/rev), the force is at its minimum level, with the basalt-reinforced composite (B_14_) having the minimum value. From the color gradients and directions of surface slopes in the figures, it is seen that the minimum levels of force occur in the basalt-reinforced composite at low feed rates. At maximum force levels, we have the G_14_ material and at high feed rates. From the figures, the hybrid composites have consistent levels of force for different input rates.

**Figure 14:**
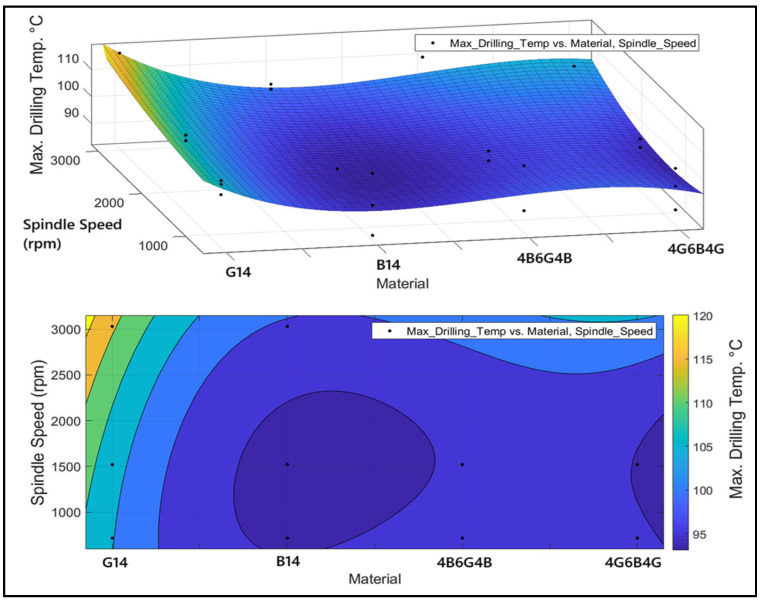
Three-dimensional surface and contour plots showing the variation of maximum drilling temperature with material type and spindle speed.

Figure 14 displays the difference in maximum temperature with respect to material type and factors of spindle speed. It was seen through the ANOVA analysis that the influence of feed rate at maximum temperature was only 0.25%. Since the rate is indicative of minimal influence, the graph focused solely on the material and the factor of spindle speed. The 3D surface graph represents the trend in temperature difference in various materials and spindle rates, and the contour graph below represents the same data in a 2D plane with the help of color gradients to represent different levels of temperature. G_14_ has the highest temperature when evaluated by material type. This is perhaps due to the heightened heat resistance offered by glass fiber, along with the increased friction from the drill bit. B_14_ has the minimum temperature due to the relatively lower thermal resistance possessed by basalt fiber and the higher penetrability offered by the drill bit to the material. Hybrid composites show moderate thermal values. As the spindle speed increases, there is a downward trend in temperature, even during the transition from low to high rates (1000 rpm to 3000 rpm). This is explained by the increased friction and temperature due to the longer duration of contact of the drill bit with the material at low spindle velocities. At high spindle speeds (approximately 3000 rpm), the temperature is reduced due to the decreased duration of contact. At high spindle speeds (3000 rpm and above), there might be temperature increases in the vicinity due to sudden thermal changes in the cutting zone. Thus, basalt-reinforced (B_14_) materials should prioritize lowering the temperature of drilling. Increasing the spindle speed tends to lower the temperature, but the appropriate speed interval should be determined to improve tool life and the efficiency of machining. Hybrid composites are suitable for various uses due to balanced thermal behavior. This work will help to lower tool wear and efficiency in the process of machining by offering key information for parameter optimization for the processes of drilling.

## 4. Conclusions

This study was expressly designed to establish transferable drilling guidelines by quantifying, through a 3 × 3 full-factorial matrix, how the feed rate, spindle speed, and laminate architecture collectively govern thrust force, thermal load and dimensional integrity in glass- (G_14_), basalt- (B_14_) and two glass-/basalt-hybrid (B_4_G_6_B_4_, G_4_B_6_G_4_) epoxy composites. Peak thrust force rose from 65.8 N for B_14_ at 0.1 mm rev^−1^ to 174.3 N for G_14_ at 0.3 mm rev^−1^, while hybrids provided intermediate loads (100–163 N) that balance glass stiffness with basalt compliance. Cutting temperature followed a similar trend, reaching 110–120 °C in G_14_ but remaining below 90 °C in both hybrids, indicating superior thermal stability.

The 36 controlled trials revealed three general laws: (i) feed rate is the dominant driver of mechanical resistance, explaining 73% of thrust force variance; whereas (ii) laminate thermal conductivity largely dictates maximum drill and workpiece temperatures, with material type accounting for 42% of their variability; and (iii) symmetric hybrid stacking (G_4_B_6_G_4_) consistently constrains entrance and exit diameters to within ±2% of nominal under all cutting conditions, demonstrating a self-balancing effect that is absent in monolithic glass or basalt laminates. Because these trends arise from orthogonal, statistically significant main effects that remained stable across the full industrial range studied (715–3030 rpm; 0.1–0.3 mm rev^−1^), they can be extrapolated to a wide variety of composite machining scenarios, providing process engineers with quantitative thresholds for minimizing delamination, tool wear and overheating in aerospace, marine, and renewable-energy structures.

The insertion of basalt layers into glass stacks reduces thrust force by half, prevents delamination, and inhibits drilling-induced overheating, providing a viable path toward prolonging tool life and maintaining hole integrity in multi-material composite structures.

Future investigations will focus on optimizing drill geometries and wear-resistant coatings specifically for hybrid laminates, mapping tool-life evolution under coolant-assisted, cryogenic and minimum-quantity-lubrication regimes, and coupling physics-based with machine-learning models to predict thrust force, temperature, and delamination concurrently; they will also examine alternative lay-ups that incorporate carbon plies or varied orientations, and extend the existing factorial framework into a multi-objective optimization that integrates energy consumption and life-cycle cost to guide sustainable manufacturing of lightweight composite assemblies.

## Figures and Tables

**Table 1:** Physical and mechanical properties of epoxy and fabrics (supplier’s data).

Materials	Fiber Architecture	Nominal Weight (g/m^2^)	Tensile Strength (MPa)	Young’s Modulus (GPa)	Density (g/cm^3^)
Epoxy	-	-	83	2900	1.13
E-Glass fabric	Plain weave	200	2306	81.50	2.59
Basalt fabric	Plain weave	200	3170	89	2.75

**Table 2:** Experimental results.

Material	Feed Rate (mm/rev)	Spindle Speed (rpm)	Thrust Force (N)	Drill Temp. (°C)	Material Temp. (°C)
G_14_	0.1	715	84.22	60.2	48.5
G_14_	0.1	1520	107.57	61.3	58.2
G_14_	0.1	3030	113.33	52	67.1
G_14_	0.2	715	112.86	51.2	42.2
G_14_	0.2	1520	133.8	49.7	45.1
G_14_	0.2	3030	134.72	42.9	50.8
G_14_	0.3	715	163.39	44.1	35.9
G_14_	0.3	1520	167.25	36.5	38
G_14_	0.3	3030	174.31	43.1	46.5
B_14_	0.1	715	65.82	39.6	36
B_14_	0.1	1520	69.06	40.9	37.1
B_14_	0.1	3030	84.04	37.3	38.8
B_14_	0.2	715	90.11	38.9	33.9
B_14_	0.2	1520	91.65	39.2	37.9
B_14_	0.2	3030	91.45	38.3	41.2
B_14_	0.3	715	133.09	36.8	35.2
B_14_	0.3	1520	137.06	38.2	39.8
B_14_	0.3	3030	146.3	35.4	44.3
B_4_G_6_B_4_	0.1	715	77.76	55.4	49.1
B_4_G_6_B_4_	0.1	1520	73.22	40.2	38.1
B_4_G_6_B_4_	0.1	3030	80.46	38.1	41.4
B_4_G_6_B_4_	0.2	715	125.75	38.6	36.8
B_4_G_6_B_4_	0.2	1520	133.43	40.8	39.2
B_4_G_6_B_4_	0.2	3030	120.17	39.7	43.7
B_4_G_6_B_4_	0.3	715	162.92	41.6	38
B_4_G_6_B_4_	0.3	1520	131.05	38.7	40.4
B_4_G_6_B_4_	0.3	3030	150.31	38.1	45.3
G_4_B_6_G_4_	0.1	715	75.89	47.4	47.6
G_4_B_6_G_4_	0.1	1520	89.01	44.5	48.4
G_4_B_6_G_4_	0.1	3030	97.48	42.3	49.6
G_4_B_6_G_4_	0.2	715	88.84	40.3	36.5
G_4_B_6_G_4_	0.2	1520	94.09	38.5	40.9
G_4_B_6_G_4_	0.2	3030	103.61	38.5	41
G_4_B_6_G_4_	0.3	715	138.25	43.8	39.2
G_4_B_6_G_4_	0.3	1520	135.83	38.9	41.2
G_4_B_6_G_4_	0.3	3030	137.22	40.4	50.7

**Table 3:** Factorial analysis results of the thrust force generated during drilling of hybrid composites.

Source of Variation	DF *	Seq SS *	Contribution *	Adj SS *	Adj MS *
Model	35	33,164.9	100.00%	33,164.9	947.6
Linear	7	30,040.3	90.58%	30,040.3	4291.5
Material	3	5144.6	15.51%	5144.6	1714.9
Feed rate (mm/rev)	2	24,339.8	73.39%	24,339.8	12169.9
Spindle speed (rpm)	2	555.8	1.68%	555.8	277.9
Two-way interactions	16	2719.7	8.20%	2719.7	170
Material X feed rate (mm/rev)	6	1573.5	4.74%	1573.5	262.3
Material X spindle speed (rpm)	6	686.8	2.07%	686.8	114.5
Feed rate (mm/rev) X spindle speed (rpm)	4	459.4	1.39%	459.4	114.8
Three-way interactions	12	404.9	1.22%	404,9	33.7
Material X feed rate (mm/rev) X spindle speed (rpm)	12	404.9	1.22%	404.9	33.7
Error	0	0	0	0	0
Total	35	33,164.9	100.00%		

* Degrees of freedom (DF), Sequential sum of squares (Seq SS), Percentage contribution (%), Adjusted sum of squares (Adj SS), and Adjusted mean square (Adj MS).

**Table 4:** ANOVA table showing the factor effects on the maximum drilling temperature (°C) during drilling of hybrid composites with the drill.

Source of Variation	DF *	Seq SS *	Contribution *	Adj SS *	Adj MS *
Model	35	2390.32	100.00%	2390.32	68.295
Linear	7	140.7	58.77%	1404.7	200.672
Material	3	991.88	41.50%	991.88	330.628
Feed rate (mm/rev)	2	6.06	0.25%	6.06	3.028
Spindle speed (rpm)	2	406.76	17.02%	406.76	203.381
Two-way interactions	16	457.81	19.15%	457.81	28.613
Material X feed rate (mm/rev)	6	164.35	6.88%	164.35	27.392
Material X spindle speed (rpm)	6	62.1	2.60%	62.1	10.351
Feed rate (mm/rev) X spindle speed (rpm)	4	231.36	9.68%	231.36	57.839
Three-way interactions	12	527.8	22.08%	527.8	43.984
Material X feed rate (mm/rev) X wpindle speed (rpm)	12	527.8	22.08%	527.8	43.984
Error	0	0	0	0	0
Total	35	2390.32	100.00%		
Source					

* Degrees of freedom (DF), Sequential sum of squares (Seq SS), Percentage contribution (%), Adjusted sum of squares (Adj SS), and Adjusted mean square (Adj MS).

## Data Availability

The datasets presented in this article are not readily available because the data are part of an ongoing study. Requests to access the datasets should be directed to the Corresponding Author.

## Abbreviations

The following abbreviations are used in this manuscript:

SEM	Scanning electron microscope
ANOVA	Analysis of Variance
